# The longitudinal association between objectively-measured school-day physical activity and academic achievement in US elementary school students

**DOI:** 10.1186/s12966-022-01328-7

**Published:** 2022-07-23

**Authors:** Paul N. Elish, Cassandra S. Bryan, Peter J. Boedeker, Hannah G. Calvert, Christi M. Kay, Adria M. Meyer, Julie A. Gazmararian

**Affiliations:** 1 Department of Epidemiology, Rollins School of Public Health, Emory University, 1518 Clifton Road, Atlanta, GA 30322 USA; 2 Department of Curriculum, Instruction, and Foundational Studies, College of Education, Boise State University, Boise, USA; 3 Center for School and Community Partnerships, College of Education, Boise State University, Boise, USA; 4 HealthMPowers, 250 Scientific Dr NW #500, Norcross, GA 30092 USA

**Keywords:** Physical activity, Academic achievement, Accelerometry, School health, Elementary students

## Abstract

**Background:**

It is recommended that school-aged children accrue 30 minutes of daily moderate-to-vigorous physical activity (MVPA) in school. Current literature is inconclusive about the long-term associations between school-based physical activity and academic achievement. In this study, we use a large sample and longitudinal design to rigorously evaluate whether school-day MVPA is associated with academic achievement.

**Methods:**

In a diverse suburban public school district, 4936 Grade 4 students were recruited in 40 elementary schools. Students wore accelerometers to measure school-day MVPA for 15 days across three semesters. Academic performance data was collected across Grade 3 fall to Grade 5 spring, including teacher-assigned grades and standardized test scores. Multilevel modeling was conducted controlling for student demographics and school characteristics.

**Results:**

Cross-sectional analyses found small negative associations in Grade 4. Grade 4 full-year mean daily school-day MVPA had β = −-0.066, β = −-0.063, β = −-0.066, and β = −-0.058 associations (*p* <  0.001) with Grade 4 math, reading, spelling, and writing grades respectively, and Grade 4 full-year mean daily school-day MVPA had β = −-0.206 and β = −-0.283 (*p* <  0.001) associations with Grade 4 math and English Language Arts (ELA) standardized test scores respectively out of approximately 500 points. Longitudinal analyses found no significant associations between Grade 4 full-year mean daily school-day MVPA and Grade 5 Fall course grades. Results also indicated small negative associations for students attaining 30+ minutes of daily school-day MVPA compared to those attaining less than 15 minutes, but only in Grade 4 Fall cross-sectional analyses where teacher-assigned reading, spelling, and writing grades were − 1.666, − 1.638, and − 1.993 points lower respectively (*p* <  0.001).

**Conclusion:**

The cross-sectional findings, while statistically significant in a negative direction, have a negligible association when translated practically. For example, even if students attained twice the recommended amount of school-day MVPA – which would constitute an approximately 300% increase from current levels – results suggest that grades would only decrease by 2 points on a 100-point scale. Furthermore, longitudinal analyses suggest school-day MVPA does not have a predictive association with course grades or standardized test scores. Findings suggest school-based MVPA implemented in accordance with recommendations does not meaningfully detract from academic progress.

**Trial registration:**

ClinicalTrials.gov, NCT03765047. Registered 05 December 2018 - Retrospectively registered, https://clinicaltrials.gov/ct2/show/NCT03765047

**Supplementary Information:**

The online version contains supplementary material available at 10.1186/s12966-022-01328-7.

## Background

While physical activity’s (PA) health benefits are well known [1–3], anywhere from half to over 70% of elementary-aged children in the United States do not reach the recommended 60 minutes of moderate-to-vigorous PA (MVPA) per day [4, 5]. Serving more than 95% of US children ages 5-17 [6], the school environment is a valuable setting to engage students in PA [7]. It is advised that children accumulate at least 30 of their 60 recommended daily MVPA minutes in schools [5], but this threshold is often unmet [8]. Particularly since the passage of the No Child Left Behind Act, school administrators have cut significant time from physical education (PE) and recess to increase time for coursework [5]. Given that academics are the clear priority for limited school time and resources, interventions focused on increasing school-day PA may be more successful if supported by evidence that increased PA improves academic achievement.

Literature on the relationship between school-day PA and academic achievement suggests either a neutral or small positive association. In 2010, a review of 50 studies indicated that increasing or maintaining school-day PA – including PE, recess, PA breaks, and other school PA practices – had great promise for improving educational outcomes, but suggested more research was needed [9]. More recently, several systematic reviews have examined the relationship between specific types of school-day PA interventions and academic achievement. A meta-analysis evaluating the impact of physically active lessons during the school day found small improvements in overall educational outcomes (effect size = 0.81; 95% CI 0.47-1.14) in elementary and preschool settings, including on standardized test scores [10]. A 2019 systematic review and metanalysis of the academic impact of “active classrooms” similarly found a small positive effect on academic performance compared to traditional, sedentary classrooms (standardized mean difference = 0.28; 95% CI: 0.09-0.47) [11]. However, other reviews have suggested a neutral association between school-day PA interventions and academic achievement, such as in a 2019 systematic review of “Active Break” school-based interventions [12].

Still other reviews have noted different associations across school subjects, with slightly stronger positive association for mathematics achievement. A 2019 systematic review found that 15 of 25 (60%) analyses from high-quality studies identified a positive relationship between school-day PA interventions and academic achievement, especially for math [13], and a meta-analysis of 26 studies enrolling 10,205 children aged 4 to 13 found small positive effect sizes for mathematics-related skills (0.21, 95% CI 0.09 to 0.33) and reading (0.13, 95% CI 0.02 to 0.24) [14]. Yet, there is also evidence of a neutral PA-achievement relationship for mathematics. Another systematic review specifically looking at school-based physical activity and mathematics performance found nearly equal numbers of studies reporting neutral and positive associations [15].

There are several limitations to existing literature on PA and academic achievement. Many studies’ samples are too small to detect effects [11, 16], and many studies have low-quality (especially cross-sectional) designs [17]. Longitudinal studies frequently have short follow-up times [18]. Inconsistent measures of PA and academic achievement could also underlie inconsistent conclusions. Many studies rely on self-reported questionnaires instead of objectively-measured PA [13, 19, 20] and do not account for PA intensity [20, 21]. As for variation in measuring academic achievement, a 2017 systematic review of classroom-based PA interventions found a positive impact on academic achievement when progress monitoring tools (e.g., short, repeated assessments) were used to measure achievement, but not when standardized tests were used. The authors hypothesized this was because standardized tests are less sensitive to small, curriculum-specific improvements [20].

To address previous limitations and inconsistent results in the existing literature, the goal of this study is to rigorously examine the longitudinal relationship between students’ school-day PA and academic outcomes. This was accomplished by conducting three investigations of the association between PA and academic achievement. First, the within-semester associations between PA and course grades (math, reading, spelling, writing) were evaluated for Grade 4 Fall, Grade 4 Spring, and Grade 5 Fall (referred to as T1, T2, and T3 respectively in this manuscript). Second, associations between Grade 4 mean MVPA and mean course grades and Grade 4 mean MVPA and standardized test scores were evaluated. Third, the longitudinal associations between PA measured in Grade 4 and course grades in Grade 5 Fall were investigated. These same analyses were then replicated with MVPA treated categorically (0-15 minutes MVPA, 15-30 minutes MVPA, 30+ minutes MVPA) to assess the potentially incremental association between minutes of MVPA and academic achievement, particularly as students approach and then exceed the recommended 30 minutes of in-school MVPA. The study uses objective accelerometer-based PA measures to examine these relationships in a racially, ethnically, and socioeconomically diverse population of 4936 Grade 4 students.

## Methods

### Study design and population

We conducted a cluster-randomized controlled trial with a total of 40 elementary schools (20 intervention; 20 control) from a large, suburban school district in the US state of Georgia. The study aimed to follow students over a two-year intervention period including Grade 4 Fall (Fall 2018; “T1”), Grade 4 Spring (Spring 2019; “T2”), Grade 5 Fall (Fall 2019; “T3”), and Grade 5 Spring (Spring 2020; “T4”), though study activities ended midway through T4 in March 2020 due to the onset of the COVID-19 pandemic. The evidence-based *Health Empowers You!* intervention [22, 23] was implemented across the entire study period from September 2018 to March 2020 with the goal of sustainably elevating students’ school-day MVPA. The intervention also ensured some students experienced higher MVPA levels closer to the recommended 30 minutes of school-day MVPA, allowing for more rigorous assessment of the relationship between school-day MVPA and academic achievement. *Health Empowers You!* is a multi-level intervention designed to shift school practices and culture to increase elementary school students’ levels of school-day PA. Trained Physical Activity Specialists (PASs) provided training and technical assistance to teachers to implement the PA intervention. Teachers received various resources to increase school-day PA, including web content, weekly calendars outlining PA resources and strategies, monthly training webinars, and exercise equipment.

Power was calculated using simulation and a Bonferroni correction to the alpha level of 0.05 given multiple hypotheses, yielding an adjusted alpha of approximately 0.0003. Specifying an unconditional intraclass correlation coefficient (ICC) of 0.25 (across the school and teacher levels) and a standardized effect size of 0.25 between PA and academic achievement based on meta-analytic reviews of the relationship, 40 schools with 6 teachers per school and 20 students per teacher gave a power of 100%.

For school recruitment, the school district provided demographic data for all the district’s elementary schools, including number of Grade 3 classes, mean number of students per Grade 3 class, racial/ethnic composition of the student body, and socioeconomic status (SES) of students’ families, which was proxied by the percentage of students who were eligible for free or reduced-priced lunch (FRL). Amount of monthly PE time at each school was also accounted for in randomization based on information from school district administrators on PE class scheduling.

To ensure that both higher SES and lower SES schools were sampled, 20 schools each were randomly selected from among the districts’ higher SES stratum schools (less than 50% of students eligible for FRL) or the lower SES stratum schools (50% or more of students eligible for FRL). Within each stratum, it was confirmed that the demographics of the 20 selected schools were comparable to the demographics of all schools in the stratum.

The 40 selected schools were then randomized to intervention or control using an urn procedure that adjusted the probabilities of allocation based on two key school-level characteristics: SES (based on FRL) and number of monthly minutes of PE scheduled for Grade 4 students. Once 20 schools were allocated to the intervention and 20 schools to control, demographic characteristics of the intervention and control groups were compared to confirm there were no statistically significant differences in characteristics between the intervention and control groups. All 40 approached schools agreed to participate in the project and accepted the condition randomization in January 2018.

All Grade 4 students not enrolled in a full-time special education classroom at participating schools at the beginning of the 2018-2019 school year were eligible for enrollment in the study. Special education teachers participated in training and received resources for implementation of the intervention at their discretion in the intervention schools, but students in special education classrooms were not included in data collection because these classes include multiple grade levels and required complex additional supports.

Information about the study was distributed to parents in August 2018 with facilitation by the principal and office staff at all participating schools. Student informed consent agreements (available in English, Spanish, Vietnamese, and several other languages) were required from participating students’ parents/guardians. Enrollment in the study included providing parental consent and student assent for participation in PA measurement via accelerometry and authorizing the school district to share archival records on standardized test scores, teacher-assigned grades, attendance, and tardiness as part of the analytic data set provided to the research team. Of 6525 Grade 4 students across the 40 schools, 4936 (76%) were enrolled in the study. Of the 4936 students, 4320 (87.5%) had a valid accelerometer measure in T1, 3800 (77.0%) in T2, and 3588 (72.7%) in T3.

The school district administration, district IRB, and Emory University IRB (IRB00095600) approved this study. School district leadership, school leadership, and teachers were extensively involved in the study’s implementation process. The school district research department reviewed and approved the proposal and study design, principals were engaged in recruitment and scheduling trainings, and district-level administration provided data and ensured smooth implementation that would not overburden schools. The *Health Empowers You!* intervention also has teachers and school administrators design a unique school activity plan that meets their school’s specific needs.

Additional details about the study’s protocol are provided in a previous manuscript [24].

### Data sources

Data sources include: (1) school district records of student academic and demographic data, and (2) ActiGraph wGT3X-BT 3-axis accelerometers (ActiGraph LLC, Pensacola, FL), attached on a waist belt. Students put on their assigned accelerometers at the beginning of the school day and removed them before leaving school. ActiLife software was used to download and score the data, and filter to only school-day minutes for scoring. Non-wear time was defined as 60 consecutive minutes of zero counts, allowing for up to 2 minutes of counts between 0 and 100 [25]. Data were collected in 15-second epochs and scored using Evenson activity threshold cut points [26].

### Measures

#### Exposure

Accelerometer-measured PA was the primary exposure. Criteria for a valid day required students to wear the accelerometer for at least 80% of the school day. Students needed at least 3 valid days of wear time during the 5-day measurement period each semester to be included in analyses for that semester. A single measure of mean daily MVPA minutes was calculated in each semester for students who met the 3-day criteria. After excluding students with insufficient accelerometer data, students included in the analysis had an average of 4.58, 4.23, and 4.52 valid days of accelerometer wear (range 3-5 days) for T1, T2, and T3, respectively, and an average 98, 96, and 98% mean daily wear time (range 82-100%, 82-100%, and 84-100%) for T1, T2, and T3.

#### Primary outcomes

Course grades and Grade 4 standardized test scores were examined as outcomes. Teachers assigned course grades each semester (T1, T2, and T3) for math, reading, spelling, and writing on a 100-point scale. The Georgia Milestone standardized test in English Language Arts (ELA) and math is administered each spring for students in grades 3 to 8. The test was first used in Georgia in 2015 and is designed to measure students’ knowledge and skills related to state-adopted content standards for each academic subject [27]. Participating students’ results from the Spring 2019 Grade 4 Georgia Milestones tests were used; participant math scale scores ranged from 394 to 715, ELA scale scores ranged from 357 to 775, and Lexile scores ranged from 190 to 1300. Course grades were not assigned and Georgia Milestones tests were not conducted in Spring 2020 due to the COVID-19 pandemic.

#### Covariates

School district data was used for student-level and school-level covariates. Student sex, race/ethnicity, physical/learning disability status, participation in special education courses, English language learner (ELL) status, FRL status, departmentalized teacher status, prior academic achievement, prior absenteeism, and prior tardiness were controlled for in all models. Student sex was either “male” or “female,” and student race/ethnicity was categorized as “Asian,” “Black,” “Hispanic,” “Mixed,” or “White.” Physical or learning disability, ELL, and FRL status were dichotomized as “yes” or “no.” Student FRL status was used as a proxy for SES. Students were eligible for FRL if their family household income was at most 185% of the federal poverty level [28]. Special education participation was incorporated as a variable ranging from zero to four based on the number of special education courses students were enrolled in across math, reading, spelling, and writing. Student prior achievement was defined as the previous year’s course grade or standardized test score, in accordance with the outcome assessed in analyses; for example, the analysis using Grade 4 Georgia Milestones math standardized test scores controlled for each student’s Grade 3 Georgia Milestones math standardized test score. Finally, a student’s prior absenteeism and prior tardiness were measured by percent days absent and tardy in 3rd grade. Some teachers were departmentalized, meaning students rotated between them and other teachers for core classes. The teacher level was not included in multi-level analyses because of student rotation across departmentalized teachers, and departmentalization entered the model instead as a student characteristic.

At the school level, analyses controlled for percentage of students who were female, Black, Hispanic, and receiving FRL, along with intervention or control status.

### Statistical analyses

Two-level random-intercepts models [29, 30] were utilized to estimate the associations of interest to account for the loss of independence of observation when lower-level units (e.g., students) are observed within higher-level units (e.g., schools). After running models with MVPA measured continuously, models were run with students’ MVPA grouped into three categories of mean daily school-day MVPA: less than or equal to 15 minutes, greater than 15 and less than or equal to 30 minutes, and greater than 30 minutes. These categories allowed assessment related to the recommendation that students attain at least 30 minutes of daily MVPA during school hours [5], specifically evaluating the difference in achievement between the low-daily-MVPA group (the <= 15 minutes category) and the group approaching the recommended 30 minutes (the 15 to 30 minute category), and between the low-daily-MVPA group and the group exceeding the 30-minute recommendation (the 30+ minutes category).

The unconditional multilevel model was used to estimate the ICC. Generally, values of the ICC above 0.05 suggest violations to the independent observations assumption and justify multilevel procedures [31]. The conditional random-intercepts model was used for estimating associations of interest. Study outcomes’ ICCs ranged from 0.117 to 0.212, which indicate a lack of independence of observation across schools and confirmed the need for multilevel modeling.

Depending on the cross-sectional analysis, the outcome was (1) the teacher-assigned semester course grade, (2) the mean of Grade 4 teacher-assigned grades from fall and spring semesters (T1 and T2), or (3) Grade 4 standardized test scores (from T2). For longitudinal analyses, a residualized change score approach was used wherein the outcome was Grade 5 teacher-assigned grades (from T3) while Grade 4 teacher-assigned grades (averaged from T1 and T2) were included as a covariate. Prior achievement entered each model grand mean centered. For categorical variables, the reference group was white males not eligible for FRL. Standardized estimates of MVPA’s coefficient were found by multiplying the coefficient by MVPA’s standard deviation and dividing by the outcome’s standard deviation [32]. To account for multiple tests, a Bonferroni adjusted critical *p*-value of 0.00271 was utilized for statistical significance.

Variables were missing data either because students were not enrolled in the participating schools for the entire study or because their observation did not meet criteria for inclusion (e.g., their accelerometer wear-time did not meet the threshold to count as a valid accelerometer measurement). Multiple imputation accounted for missing data. Twenty imputed datasets were created using the multilevel multiple imputation program Blimp [33]. Implausible imputed values were set to variables’ upper or lower bounds. Descriptive statistics were run on the non-imputed data. Final estimates of fixed and random effects were calculated using Rubin’s rules [34].

## Results

### Descriptive analysis of study population

Analyses include data from a majority of the 4936 students enrolled in the study. The requirement that a student be enrolled for at least one semester of analysis reduced the analytic sample to between 4189 and 4869, depending on the analysis. The sample was diverse, with 12.2% of the sample identifying as Asian, 25.2% Black, 33.2% Hispanic, 4.3% Mixed and 24.8% White (Table 1). Approximately 53.1% received FRL, and 50.0% were female. About a third of participants were current or former ELL (35.1%) and 12.9% had learning or physical disabilities. Mean course grades were similar across the sample for Grade 3, Grade 4, and Grade 5 fall. Mean standardized test scores rose across Grades 3 and 4 in math, ELA, and Lexile. Mean daily school-day MVPA declined over the study period. Students on average had 21.14 minutes of MVPA in T1, 21.85 minutes in T2, and 18.91 minutes in T3. Variables’ missingness is described in Table 1.

**Table 1:** Student and school demographics, academic achievement, and physical activity data, Grades 4 to 5

Student-level data, ***n =*** 4936 ^**a**^			
**Variable**	**Count / mean**	**% / SD**	**% missing data**
**Sex**			
Female	2466	(50.0%)	0
Male	2465	(49.9%)	
**Race/Ethnicity**			
Asian	601	(12.2%)	0.1
Black	1243	(25.2%)	
Hispanic	1640	(33.2%)	
Mixed	213	(4.3%)	
White	1226	(24.8%)	
**Free/Reduced-Price Lunch Recipient**			
Yes	2622	(53.1%)	0.1
No	2309	(46.8%)	
**English Language Learner**			
Yes	1735	(35.1%)	0.1
No	3196	(64.7%)	
**Student With Disabilities**			
Yes	637	(12.9%)	0.1
No	4294	(87.0%)	
**Grade 3 Absences (%)**	3.1	(3.0)	0.1
**Grade 3 Tardies (%)**	1.9	(3.6)	0.1
**Special Education Participant**			
Yes	242	(4.9%)	0
No	4694	(95.1%)	
**Treatment Group**			
Intervention	2621	(53.1%)	0
Control	2315	(46.9%)	
**Grade 3 Mean Course Grades**			
Math	83.5	(9.4)	8.4
Reading	82.9	(9.0)	8.4
Spelling	87.9	(9.0)	12.2
Writing	84.4	(7.8)	9.1
**Grade 4 Fall (T1) Course Grades**			
Math	81.5	(12.0)	2.1
Reading	80.9	(10.3)	2.0
Spelling	86.7	(11.3)	5.4
Writing	83.2	(8.9)	2.0
**Grade 4 Spring (T2) Course Grades**			
Math	83.2	(10.6)	3.3
Reading	81.8	(9.9)	3.0
Spelling	87.3	(10.2)	5.2
Writing	83.8	(8.7)	3.3
**Grade 5 Fall (T3) Course Grades**^**b**^			
Math	82.0	(11.5)	15.1
Reading	82.4	(9.4)	14.4
Spelling	87.6	(9.6)	16.5
Writing	84.9	(8.2)	15.0
**Grade 3 Standardized Test Scores**^**c**^			
Math	541.4	(49.9)	6.0
English Language Arts	527.1	(58.2)	6.0
Lexile	728.6	(219.6)	6.0
**Grade 4 (T2) Standardized Test Scores**^**c**^			
Math	549.2	(53.7)	3.3
English Language Arts	535.2	(55.5)	3.3
Lexile	897.2	(221.1)	3.3
**Mean daily school-day moderate-to-vigorous physical activity (MVPA) minutes**			
T1	21.1	(9.2)	12.5
T2	21.9	(10.0)	23.0
T3	18.9	(8.9)	27.3
**Mean valid days of school-day accelerometer data by semester**			
T1	4.58	(0.65)	12.5
T2	4.23	(0.73)	23.0
T3	4.52	(0.67)	27.3
**Mean percent accelerometer wear time by semester (%)**			
T1	98.5	(2.1)	12.5
T2	96.4	(4.6)	23.0
T3	98.3	(2.3)	27.3
**Minutes of mean daily school-day MVPA, categorical, T1**^**d**^			
< =15	1247	(25.7%)	12.5
15 < x < =30	2856	(58.9%)	
30+	748	(15.4%)	
**Minutes of mean daily school-day MVPA, categorical, T2**^**d**^			
< =15	1255	(25.9%)	23.0
15 < x < =30	2628	(54.2%)	
30+	968	(20.0%)	
**Minutes of mean daily school-day MVPA, categorical, T3**^**d**^			
< =15	1532	(36.2%)	27.3
15 < x < =30	2248	(53.1%)	
30+	453	(10.7%)	
**School-level data (*n =*** **40)**			
**Variables**	**% / mean**	**% / SD**	**% missing data**
**School % Female**	48.6%	(4.2)	0
**School % Black**	27.3%	(12.1)	0
**School % Hispanic**	32.2%	(19.8)	0
**School % FRL**	56.1%	(26.7)	0
**Teacher Departmentalization**			
Yes	133	(46.5%)	0
No	153	(53.5%)	

^a^Not all tabulations add to 4936 due to missing data

^b^Due to COVID-related disruptions, Grade 5 Spring course grades are not incorporated in analyses

^c^Due to COVID-related disruptions, no standardized tests were conducted in Grade 5

^d^Values from imputed data

### Intervention impact on MVPA

The intervention was successful in its goal of consistently elevating intervention students’ school-day MVPA across the study period. The intervention increased school-day MVPA among intervention students relative to control students by 3 minutes in T1 and nearly 5 minutes in T2 (Table 2). The gap widened to over 5 minutes daily in T3 as school-day MVPA fell more sharply among control students.

**Table 2:** Mean daily MVPA minutes by semester and school randomization status

	School Randomization Status		
Semester	Intervention	Control	***p-***value
	Mean (SD)	Mean (SD)	
Grade 4 Fall (T1)	22.6 (9.5)	19.6 (8.5)	**< 0.001**
Grade 4 Spring (T2)	24.1 (10.4)	19.6 (9.1)	**< 0.001**
Grade 5 Fall (T3)	21.5 (9.0)	16.3 (8.0)	**< 0.001**

### Association between continuous MVPA and AA

#### Within semester

The conditional association between continuous mean daily MVPA and course grades was assessed within each semester (Table 3). For all results, the association’s coefficient was negative. T1 MVPA was significantly associated with all course grades (*β*_*Math*_ = − 0.055, *β*_*Reading*_ = − 0.052, *β*_*Spelling*_ = − 0.069, *β*_*Writing*_ = − 0.059, all *p*-values = <.0001), but T2 MVPA was only significantly associated with math course grades (*β*_*Math*_ = − 0.053, *p-*value = <.0001) and T3 MVPA was not significantly associated with any course grades.

**Table 3:** Mean daily MVPA and course grades, adjusted results^a^

Academic outcome	Coefficient (SE)	Standardized Effect Size	***p-***value
**Grade 4 Fall (T1) Course Grades**			
Math	− 0.055 (0.016)	− 0.042	**< 0.001**
Reading	−0.052 (0.013)	− 0.046	**< 0.001**
Spelling	−0.069 (0.017)	− 0.056	**< 0.001**
Writing	− 0.059 (0.013)	− 0.061	**< 0.001**
**Grade 4 Spring (T2) Course Grades**			
Math	−0.053 (0.014)	− 0.050	**< 0.001**
Reading	−0.033 (0.013)	−0.033	0.009
Spelling	−0.029 (0.017)	−0.029	0.098
Writing	−0.033 (0.013)	−0.038	0.011
**Grade 5 Fall (T3) Course Grades**			
Math	− 0.002 (0.016)	−0.002	0.922
Reading	−0.007 (0.013)	− 0.007	0.603
Spelling	− 0.016 (0.016)	−0.015	0.321
Writing	−0.013 (0.013)	− 0.014	0.353

^a^Models adjusted for student sex, race/ethnicity, FRL status, English Language Learner status, student with disabilities status, Grade 4% of days absent, Grade 4% of days tardy, departmentalization, special education course enrollment, school percentage female, school percentage black, school percentage Hispanic, school percentage FRL, school cohort (intervention or control), and prior achievement for the specific academic outcome (e.g., when assessing math grade as outcome, used Grade 3 mean math grade). In bold are the *p*-values that were statistically significant after comparing to a Bonferroni adjusted p-critical of 0.00271

#### Within grade 4 year

Results from multilevel models showed that Grade 4 mean school-day MVPA across T1 and T2 was a statistically significant and negative predictor of all Grade 4 mean course grades across T1 and T2 (*β*_*Math*_ = − 0.066, *β*_*Reading*_ = − 0.063, *β*_*Spelling*_ = − 0.066, *β*_*Writing*_ = − 0.058, all *p*-values = <.0001) and standardized test scores from T2 (*β*_*Math*_ = − 0.206, *β*_*ELA*_ = − 0.283, all *p-*values = <.0001) except Lexile (Table 4).

**Table 4:** Grade 4 mean (T1 and T2) daily MVPA and Grade 4 academic achievement, adjusted results^a^

Academic outcome	Coefficient (SE)	Standardized Effect Size	***p-***value
**Grade 4 Year (Mean T1 and T2) Course Grades**			
Math	−0.066 (0.015)	− 0.051	**< 0.001**
Reading	−0.063 (0.014)	− 0.056	**< 0.001**
Spelling	−0.066 (0.016)	−0.056	**< 0.001**
Writing	−0.058 (0.013)	−0.059	**< 0.001**
**Grade 4 (T2) Standardized Test Scores**			
Math	−0.206 (0.062)	−0.032	**< 0.001**
English Language Arts	−0.283 (0.070)	−0.043	**< 0.001**
Lexile	−0.951 (0.321)	− 0.036	0.003

^a^Models adjusted for student sex, race/ethnicity, FRL status, English Language Learner status, student with disabilities status, Grade 4% of days absent, Grade 4% of days tardy, departmentalization, special education course enrollment, school percentage female, school percentage black, school percentage Hispanic, school percentage FRL, school cohort (intervention or control), and prior achievement for the specific academic outcome (e.g., when assessing math grade as outcome, used Grade 3 mean math grade). In bold are the *p*-values that were statistically significant after comparing to a Bonferroni adjusted p-critical of 0.00271

#### Residualized change from 4th to grade 5

The association between T3 course grades and mean school-day MVPA across Grade 4 (T1 and T2) was evaluated while controlling for Grade 4 (T1 and T2) mean course grade. Findings indicated that the conditional associations were again negative yet nonsignificant for all outcomes (Table 5).

**Table 5:** Residualized course grades change from Grade 4 to Grade 5 fall (T3) predicted by Grade 4 mean (T1 and T2) daily MVPA, adjusted results^a^

Academic outcome	Coefficient (SE)	Standardized Effect Size	***p-***value
**Grade 5 Fall (T3) Course Grades**			
Math	−0.011 (0.017)	− 0.008	0.503
Reading	−0.025 (0.014)	−0.023	0.082
Spelling	−0.002 (0.018)	−0.002	0.901
Writing	−0.034 (0.014)	−0.035	0.018

^a^Models adjusted for student sex, race/ethnicity, FRL status, English Language Learner status, student with disabilities status, Grade 4% of days absent, Grade 4% of days tardy, departmentalization, special education course enrollment, school percentage female, school percentage black, school percentage Hispanic, school percentage FRL, school cohort (intervention or control), and prior achievement for the specific academic outcome (e.g., when assessing math grade as outcome, used Grade 4 mean math grade). No coefficients were statistically significant after comparing to a Bonferroni adjusted p-critical of 0.00271

### Association between categorical MVPA and AA

#### Within semester

The conditional association between categorical mean daily MVPA and course grades was assessed within each semester (Tables 6 and 7). In T1, students attaining greater than 30 minutes of mean daily school-day MVPA had reading, spelling, and writing grades that were 1.67, 1.64, and 1.99 points lower, on average, than grades for students attaining less than 15 minutes mean daily school-day MVPA (all *p*-values = <.0001). In T2 and T3, there were no significant differences in average academic achievement between students attaining 30+ minutes of mean daily school-day MVPA and students attaining less than 15 minutes mean daily school-day MVPA. There were no significant differences in academic achievement between students attaining 15-30 minutes average school-day MVPA and students attaining less than 15 minutes across T1, T2, and T3.

**Table 6:** Categorical daily MVPA and course grades, adjusted results^a^

Academic outcome and Categorical MVPA	Coefficient	Standardized Effect Size	***p-***value
**Grade 4 Fall (T1) Course Grades**			
**Math**			
15 < MVPA < 30	− 0.479	− 0.040	0.112
MVPA > 30	− 1.290	− 0.107	0.005
**Reading**			
15 < MVPA < 30	− 0.507	− 0.049	0.054
MVPA > 30	− 1.666	− 0.162	**< 0.001**
**Spelling**			
15 < MVPA < 30	− 0.424	− 0.048	0.092
MVPA > 30	− 1.638	− 0.184	**< 0.001**
**Writing**			
15 < MVPA < 30	− 0.480	− 0.043	0.170
MVPA > 30	− 1.993	− 0.176	**< 0.001**
**Grade 4 Spring (T2) Course Grades**			
**Math**			
15 < MVPA < 30	− 0.481	− 0.045	0.103
MVPA > 30	− 1.216	− 0.115	0.005
**Reading**			
15 < MVPA < 30	− 0.171	−0.017	0.540
MVPA > 30	−0.700	− 0.071	0.068
**Spelling**			
15 < MVPA < 30	− 0.290	− 0.033	0.277
MVPA > 30	− 0.791	−0.091	0.028
**Writing**			
15 < MVPA < 30	−0.439	− 0.043	0.163
MVPA > 30	− 0.871	− 0.085	0.053
**Grade 5 Fall (T3) Course Grades**			
**Math**			
15 < MVPA < 30	0.288	0.025	0.319
MVPA > 30	−0.570	− 0.050	0.234
**Reading**			
15 < MVPA < 30	0.082	0.009	0.733
MVPA > 30	−0.417	−0.044	0.283
**Spelling**			
15 < MVPA < 30	−0.110	− 0.013	0.641
MVPA > 30	− 0.674	− 0.082	0.096
**Writing**			
15 < MVPA < 30	− 0.171	− 0.018	0.553
MVPA > 30	− 1.031	− 0.107	0.049

^a^Models adjusted for student sex, race/ethnicity, FRL status, English Language Learner status, student with disabilities status, Grade 4% of days absent, Grade 4% of days tardy, departmentalization, special education course enrollment, school percentage female, school percentage black, school percentage Hispanic, school percentage FRL, school cohort (intervention or control), and prior achievement for the specific academic outcome (e.g., when assessing math grade as outcome, used Grade 3 mean math grade). In bold are the *p*-values that were statistically significant after comparing to a Bonferroni adjusted p-critical of 0.00271

**Table 7:** Grade 4 mean (T1 and T2) daily MVPA and Grade 4 academic achievement, adjusted results^a^

Academic outcome	Coefficient	Standardized Effect Size	***p-***value
**Grade 4 Year (Mean T1 and T2) Course Grades**			
**Math**			
15 < MVPA < 30	− 0.176	− 0.016	0.596
MVPA > 30	−1.011	− 0.093	0.042
**Reading**			
15 < MVPA < 30	− 0.284	− 0.030	0.270
MVPA > 30	−0.979	− 0.102	0.018
**Spelling**			
15 < MVPA < 30	− 0.385	− 0.046	0.315
MVPA > 30	−1.308	− 0.158	0.515
**Writing**			
15 < MVPA < 30	− 0.365	− 0.037	0.172
MVPA > 30	−1.326	− 0.134	0.003
**Grade 4 (T2) Standardized Test Scores**			
**Math**			
15 < MVPA < 30	−3.446	− 0.064	0.005
MVPA > 30	−5.790	− 0.108	0.004
**English Language Arts**			
15 < MVPA < 30	−1.204	−0.022	0.050
MVPA > 30	−4.062	−0.073	0.029
**Lexile**			
15 < MVPA < 30	−2.205	−0.010	0.039
MVPA > 30	−10.177	−0.046	0.029

^a^Models adjusted for student sex, race/ethnicity, FRL status, English Language Learner status, student with disabilities status, Grade 4% of days absent, Grade 4% of days tardy, departmentalization, special education course enrollment, school percentage female, school percentage black, school percentage Hispanic, school percentage FRL, school cohort (intervention or control), and prior achievement for the specific academic outcome (e.g., when assessing math grade as outcome, used Grade 3 mean math grade). In bold are the *p*-values that were statistically significant after comparing to a Bonferroni adjusted p-critical of 0.00271

#### Within grade 4 year

Results from multilevel models showed that categorical Grade 4 mean school-day MVPA (T1 and T2) was not significantly associated with mean course grades (T1 and T2) or standardized test scores measured at the end of the school year (T2).

#### Residualized change from 4th to grade 5

The association between Grade 5 (T3) course grades and categorical mean school-day MVPA across Grade 4 (T1 and T2) was evaluated while controlling for Grade 4 (T1 and T2) mean course grade. Findings indicated that the conditional associations were again negative yet nonsignificant for all outcomes (Table 8).

**Table 8:** Residualized course grades change from Grade 4 to Grade 5 fall (T3) predicted by Grade 4 mean (T1 and T2) daily MVPA, adjusted results^a^

Academic outcome	Coefficient	Standardized Effect Size	***p-***value
**Grade 5 Fall (T3) Course Grades**			
**Math**			
15 < MVPA < 30	−0.098	− 0.008	0.758
MVPA > 30	− 0.303	− 0.026	0.544
**Reading**			
15 < MVPA < 30	− 0.337	− 0.036	0.200
MVPA > 30	−0.718	− 0.076	0.067
**Spelling**			
15 < MVPA < 30	− 0.439	− 0.054	0.081
MVPA > 30	− 0.771	− 0.094	0.042
**Writing**			
15 < MVPA < 30	−0.084	− 0.009	0.795
MVPA > 30	− 0.148	− 0.015	0.752

^a^Models adjusted for student sex, race/ethnicity, FRL status, English Language Learner status, student with disabilities status, Grade 4% of days absent, Grade 4% of days tardy, departmentalization, special education course enrollment, school percentage female, school percentage black, school percentage Hispanic, school percentage FRL, school cohort (intervention or control), and prior achievement for the specific academic outcome (e.g., when assessing math grade as outcome, used Grade 3 mean math grade). In bold are the *p-*values that were statistically significant after comparing to a Bonferroni adjusted p-critical of 0.00271

## Discussion

Findings suggest school-based MVPA does not have a positive association with academic achievement. However, school-based MVPA also does not appear to meaningfully harm academic achievement. Longitudinal analyses examining the association between continuous Grade 4 (T1 and T2) mean school-day MVPA and Grade 5 (T3) course grades found no significant association between school-day MVPA and academic achievement after adjusting for student sex, race/ethnicity, FRL status, ELL status, disabilities status, attendance, tardiness, departmentalization, special education course enrollment, school demographic characteristics, school intervention/control status, and prior achievement. Although cross-sectional analyses using continuous MVPA produced statistically significant negative coefficients after adjusting for covariates, the associations are negligible when translated practically. For example, within-semester analyses show a 10-minute difference in mean school-day MVPA is associated with a difference of only 0.55 points in T1 math course grade. Even if students’ mean school-day MVPA reached double the recommended 30 daily minutes in school – which would constitute an approximately 300% increase from current levels – results suggest that grades would only decrease by about 2 points on a 100-point scale. The magnitudes of within-Grade-4 findings adjusted for covariates are also insignificant in practical terms. A 10-minute increase in mean school-day MVPA was associated with 0.58 to 0.66 point lower Grade 4 mean course grades on a 100-point scale, and math and ELA standardized test scale scores that were about 2.5 points lower on a 500-point scale. These standardized test score differences are equivalent to a difference of about 0.03 standard deviations for math scores and 0.04 standard deviations for ELA scores.

Analyses treating MVPA categorically to assess the academic impact of students attaining the recommended 30 minutes of daily school-day MVPA similarly did not find a meaningful difference in average achievement across MVPA categories. Longitudinal analyses found no significant difference in average achievement across MVPA categories after adjusting for covariates. Cross-sectional analyses only found significant associations for T1. During that semester, students exceeding the recommended 30 minutes of mean daily school-day MVPA had reading, spelling, and writing grades that were 1.7, 1.6, and 2.0 points lower than students attaining less than 15 minutes of mean daily school-day MVPA on a 100-point scale. The lack of significant differences in academic achievement between students attaining less than 15 minutes daily school-day MVPA and students attaining 15-30 minutes further suggests that getting students closer to the recommended 30 minutes of daily school-day MVPA does not meaningfully detract from academic progress.

In the same cohort, separate analyses found a positive indirect association from MVPA to AA through cardiorespiratory fitness. A 10-minute increase in daily school-day MVPA had a significant positive indirect effect through higher cardiorespiratory fitness associated with 0.15, 0.12, and 0.10-point increases in math, writing, and spelling grades respectively. The finding of a positive relationship between cardiorespiratory fitness and academic achievement aligns with existing literature [35–37].

The lack of a meaningful association between MVPA and academic achievement in this study deviates from some prior research suggesting a positive relationship between school-day PA and academic achievement. However, it is worth noting that prior reviews identifying a positive association have noted the association’s magnitude was small [10, 11, 14]. Furthermore, some prior systematic reviews evaluating school-day PA have found no impact on academic achievement,^12^while others have found a balance of evidence suggesting a null or slightly positive relationship [15, 38].

The findings align with previous studies incorporating objectively-measured PA. One 2018 review examined 11 studies that assessed the relationship between objectively-measured PA (both in and outside school) and academic achievement. Four high-quality studies found partial evidence for a positive relationship, six found no significant association, and one high-quality, large-sample study found a small negative association [36]. The four cross-sectional studies conducted in the US found no association among children in grades 4 to 6 (mean age 10.5 years), no association among children in grades 2 to 3 (mean age 7.8 years), no association among children of mean age 8.6 years, and a positive association only for math among students in grades 2 to 3 (mean age 7.6 years). The sole longitudinal study examined was a UK cohort study that followed students from age 11 to 16 years, which found that total PA time was negatively associated with academic achievement, but the percentage of time spent in MVPA positively predicted English achievement after controlling for total PA time and other confounders [39]. In contrast to inconsistent findings from studies with objectively-measured PA, self-reported PA was more consistently positively associated with academic achievement [36], which might account for systematic reviews that have found evidence for a positive association between PA and academic achievement [38].

In light of school-day MVPA’s non-meaningful association with academic achievement and the positive association between cardiorespiratory fitness and academic achievement, concerns that increasing school-day PA meaningfully detracts from students’ academic progress do not appear well founded. Regardless, schools can increase MVPA in ways that do not compete with other academic subjects, such as during recess, in enhanced PE classes, and through “academic accelerators” that incorporate academic content into PA. In this sample, a 10-minute increase in daily school-day MVPA would meet the recommendation for 30 minutes of school-day MVPA and contribute to students’ physical and mental health without meaningfully impacting academic achievement.

Despite the non-meaningful association between school-day PA and academic achievement, there is strong evidence for the association between PA and obesity and other health outcomes [40]. There has been a documented rise in child obesity during the COVID-19 pandemic. A cohort of 432,302 US infants, children, and adolescents aged 2-19 years experienced a doubled rate of BMI increase during the pandemic compared to a pre-pandemic period, and younger school-aged children saw the largest increases [41]. Schools across the US shifted to online and hybrid learning during the pandemic, and it is thought that lack of access to structured physical activity in school settings was one contributing factor to accelerated weight gain [41]. This highlights the importance of school-based PA that can improve children’s health without meaningfully impacting academic achievement.

### Strengths

This is the largest assessment of the association between objectively-measured PA and academic achievement yet conducted in the United States and one of the largest conducted globally. Previous studies of this association in the U.S. have enrolled fewer than 700 participants and had cross-sectional designs. Despite ending early due to COVID-related disruptions, the study’s multi-year follow up is a strength. The use of accelerometry provides a more valid measure than self-reported PA because it is not vulnerable to recall and social desirability bias. The sample diversity allows for inferences about a range of racial, ethnic, and socioeconomic groups. The high consent rate of 76% across all Grade 4 students at baseline also reduces the risk of selection bias.

### Limitations

Despite these strengths, there are at least five limitations. First, the study did not include measures of academic behaviors (e.g., time on task), which might mediate the relationship between PA and academic achievement; validly measuring these behaviors was deemed unfeasible in such a large sample. Second, students who contributed sufficient accelerometer data for analysis tended to be of higher SES and were more likely to be Asian or white than the overall sample. However, this limitation was addressed through multiple imputation. Third, the extrapolation of 3-5 days of accelerometer data to a full semester of activity levels may have been less accurate than a longer accelerometer measurement period [42]. However, even though at least 4 days of wear time is typically recommended for reliable PA estimates in children [43, 44], school-day PA is less variable than full-day data [42]. Fourth, no Grade 5 standardized tests were conducted, and follow-up time ended one semester early because of COVID-related disruptions. Finally, this study only examined school-day MVPA rather than full-day MVPA. It was not feasible to use accelerometers to objectively-measure full-day MVPA longitudinally in this large study sample.

## Conclusions

Although results do not suggest increasing school-day PA will improve student academic performance, the findings reduce concerns about school-based PA detracting from long-term academic progress. Schools should be considered appropriate venues for PA promotion, which can support children’s overall physical, mental, and emotional development. Future research in this area should include measures of student academic behaviors (e.g., time on task) to better understand mechanisms for improving academic achievement and should consider longer longitudinal designs to better understand the long-term impact of consistent PA on academic achievement. Additional studies in different school districts are also warranted to investigate how this relationship might differ across different populations and school administrative environments. Further analyses from this study population will explore mediators between school-day PA and academic achievement, including cardiorespiratory fitness and body mass index.

This study is the largest assessment of the association between objectively-measured school-based PA and academic achievement yet recorded in the United States. There is a need to increase PA among children to reap physical and mental health benefits. While findings do not indicate a positive impact of school-based MVPA on academic achievement, results suggest that physical activity interventions can be implemented in school settings without meaningfully detracting from students’ academic growth.

## Supplementary Information

**Additional file 1.**

## Data Availability

No data are available per data use agreement with participating school district.

## Abbreviations

PA: Physical activity
MVPA: Moderate-to-vigorous physical activity
PE: Physical education
PAS: Physical activity specialist
ICC: Intraclass correlation coefficient
SES: Socioeconomic status
ELA: English Language Arts
ELL: English language learner

## References

[1] Bogden JF. *Fit, Healthy, and Ready to Learn*: A school health policy guide. Part I: physical activity, health eating, and tobacco-use prevention: National Association of State Boards of Education; 2000.

[2] U.S (2008). Department of Health and Human Services. *2008 physical activity guidelines for Americans*.

[3] U.S. Department of Health and Human Services and U.S. Department of Education. Promoting better health for young people through physical activity and sports. A Report *to the President from the Secretary of Health and Human Services and the Secretary of Education.*: Centers for Disease Control and Prevention (DHHS/PHS); President's Council on Physical Fitness and Sports; Office of Elementary and Secondary Education;2000.

[4] 2018 National Survey of Childrens Health. 2020. https://www.childhealthdata.org/browse/survey/results?q=7620&r=1&g=791. Accessed 5 Feb 2021.

[5] Kohl HW, Cook HD, Institute of Medicine (2013). Educating the student body: taking physical activity and physical education to school.

[6] Centers for Disease Control and Prevention. CDC Healthy Schools. Centers for Disease Control and Prevention. https://www.cdc.gov/healthyschools/about.htm. Published 2019. Accessed 24 Aug 2020.

[7] Centers for Disease Control and Prevention. Infographic: Benefits of School-Based Physical Activity 2021. https://www.cdc.gov/healthyschools/physicalactivity/school_pa_benefits.htm.

[8] Turner L, Johnson TG, Calvert HG, Chaloupka FJ (2017). Stretched too thin? The relationship between insufficient resource allocation and physical education instructional time and assessment practices. Teach Teach Educ.

[9] Centers for Disease Control and Prevention (2010). The association between school based physical activity, including physical education, and academic performance.

[10] Norris E, van Steen T, Direito A, Stamatakis E (2020). Physically active lessons in schools and their impact on physical activity, educational, health and cognition outcomes: a systematic review and meta-analysis. Br J Sports Med.

[11] Bedard C, St John L, Bremer E, Graham JD, Cairney J (2019). A systematic review and meta-analysis on the effects of physically active classrooms on educational and enjoyment outcomes in school age children. PLoS One.

[12] Masini A, Marini S, Gori D, Leoni E, Rochira A, Dallolio L (2020). Evaluation of school-based interventions of active breaks in primary schools: a systematic review and meta-analysis. J Sci Med Sport.

[13] Singh AS, Saliasi E, van den Berg V (2019). Effects of physical activity interventions on cognitive and academic performance in children and adolescents: a novel combination of a systematic review and recommendations from an expert panel. Br J Sports Med.

[14] Álvarez-Bueno C, Pesce C, Cavero-Redondo I, Sánchez-López M, Garrido-Miguel M, Martínez-Vizcaíno V. Academic achievement and physical activity: a Meta-analysis. Pediatrics. 2017;140(6). 10.1542/peds.2017-1498 29175972

[15] Sneck S, Viholainen H, Syväoja H (2019). Effects of school-based physical activity on mathematics performance in children: a systematic review. Int J Behav Nutr Phys Act.

[16] Wassenaar TM, Williamson W, Johansen-Berg H (2020). A critical evaluation of systematic reviews assessing the effect of chronic physical activity on academic achievement, cognition and the brain in children and adolescents: a systematic review. Int J Behav Nutr Phys Act.

[17] Donnelly JE, Hillman CH, Castelli D (2016). Physical activity, fitness, cognitive function, and academic achievement in children: a systematic review. Med Sci Sports Exerc.

[18] Norris E, Shelton N, Dunsmuir S, Duke-Williams O, Stamatakis E (2015). Physically active lessons as physical activity and educational interventions: a systematic review of methods and results. Prev Med.

[19] Vetter M, Orr R, O'Dwyer N, O'Connor H (2020). Effectiveness of active learning that combines physical activity and math in schoolchildren: a systematic review. J Sch Health.

[20] Watson A, Timperio A, Brown H, Best K, Hesketh KD (2017). Effect of classroom-based physical activity interventions on academic and physical activity outcomes: a systematic review and meta-analysis. Int J Behav Nutr Phys Act.

[21] Sember V, Jurak G, Kovač M, Morrison SA, Starc G (2020). Children's physical activity, academic performance, and cognitive functioning: a systematic review and meta-analysis. Front Public Health.

[22] United States Department of Agriculture. Health EmPowers You! SNAP-Ed Library Web site. https://snaped.fns.usda.gov/library/materials/health-empowers-you. Published 2015. Accessed July 24, 2021.

[23] HealthMPowers. Program. https://healthempowersyou.org/about/. Published 2021. Accessed July 24, 2021.

[24] Boedeker P, Turner L, Calvert H (2021). Study protocol for testing the association between physical activity and academic outcomes utilizing a cluster-randomized trial. Contemp Clin Trials Commun.

[25] Troiano RP, Berrigan D, Dodd KW, Mâsse LC, Tilert T, McDowell M (2008). Physical activity in the United States measured by accelerometer. Med Sci Sports Exerc.

[26] Evenson KR, Catellier DJ, Gill K, Ondrak KS, McMurray RG (2008). Calibration of two objective measures of physical activity for children. J Sports Sci.

[27] Georgia Department of Education. Georgia Milestones Assessment System. https://www.gadoe.org/Curriculum-Instruction-and-Assessment/Assessment/Pages/Georgia-Milestones-Assessment-System.aspx. Published 2021. Accessed July 24, 2021.

[28] Feeding America. The National School Lunch Program (NSLP). https://www.feedingamerica.org/take-action/advocate/federal-hunger-relief-programs/national-school-lunch-program. Published 2021. Accessed July 10, 2021.

[29] Raudenbush SW, Bryk AS. Hierarchical linear models: Applications and data analysis methods. 2002;1:sage.

[30] Boedeker P (2017). Hierarchical linear modeling with maximum likelihood, restricted maximum likelihood, and fully Bayesian estimation. Pract Assess Res Eval.

[31] Kreft I, de Leeuw J. Introducing multilevel modeling. Sage. 1998.

[32] Snijders TA, Bosker RJ. Multilevel analysis: an introduction to basic and advanced multilevel modeling. Sage. 2011.

[33] Enders CK, Keller BT, Levy R (2018). A fully conditional specification approach to multilevel imputation of categorical and continuous variables. Psychol Methods.

[34] Rubin DB, Schenker N (1991). Multiple imputation in health-are databases: an overview and some applications. Stat Med.

[35] Santana CCA, Azevedo LB, Cattuzzo MT, Hill JO, Andrade LP, Prado WL (2017). Physical fitness and academic performance in youth: a systematic review. Scand J Med Sci Sports.

[36] Marques A, Santos DA, Hillman CH, Sardinha LB (2018). How does academic achievement relate to cardiorespiratory fitness, self-reported physical activity and objectively reported physical activity: a systematic review in children and adolescents aged 6-18 years. Br J Sports Med.

[37] Álvarez-Bueno C, Hillman CH, Cavero-Redondo I, Sánchez-López M, Pozuelo-Carrascosa DP, Martínez-Vizcaíno V (2020). Aerobic fitness and academic achievement: a systematic review and meta-analysis. J Sports Sci.

[38] Barbosa A, Whiting S, Simmonds P, Scotini Moreno R, Mendes R, Breda J. Physical activity and academic achievement: an umbrella review. Int J Environ Res Public Health. 2020;17(16). 10.3390/ijerph17165972 PMC7460146 32824593

[39] Booth JN, Leary SD, Joinson C (2014). Associations between objectively measured physical activity and academic attainment in adolescents from a UK cohort. Br J Sports Med.

[40] Centers for Disease Control and Prevention. Physical Activity Guidelines for Americans 2018.

[41] Lange SJ, Kompaniyets L, Freedman DS (2021). Longitudinal trends in body mass index before and during the COVID-19 pandemic among persons aged 2-19 years - United States, 2018-2020. MMWR Morb Mortal Wkly Rep.

[42] Fairclough SJ, Butcher ZH, Stratton G (2007). Whole-day and segmented-day physical activity variability of Northwest England school children. Prev Med.

[43] Trost SG, McIver KL, Pate RR (2005). Conducting accelerometer-based activity assessments in field-based research. Med Sci Sports Exerc.

[44] Barreira TV, Schuna JM, Tudor-Locke C (2015). Reliability of accelerometer-determined physical activity and sedentary behavior in school-aged children: a 12-country study. Int J Obes Suppl.

