# Supplementary material for: The longitudinal association between objectively-measured school-day physical activity and academic achievement in US elementary school students
Source: Int J Behav Nutr Phys Act. 2022 Jul 23;19:90. doi: 10.1186/s12966-022-01328-7 (PMC9308117; doi:10.1186/s12966-022-01328-7)
Supplement: Supplementary file 1 — Additional file 1. [file 12966_2022_1328_MOESM1_ESM.docx]

**Table 9.** Unadjusted associations, mean daily MVPA and academic achievement

| **Mean daily MVPA and course grades (within semester), unadjusted results^a^** | | |
| --- | --- | --- |
| **Academic outcome** | **Coefficient (SE)** | **p-value** |
| **Grade 4 Fall (T1) Course Grades** |  |  |
| Math | -0.139 (0.019) | **< 0.001** |
| Reading | -0.143 (0.017) | **< 0.001** |
| Spelling | -0.133 (0.015) | **< 0.001** |
| Writing | -0.125 (0.019) | **< 0.001** |
| **Grade 4 Spring (T2) Course Grades** |  |  |
| Math | -0.142 (0.018) | **< 0.001** |
| Reading | -0.135 (0.016) | **< 0.001** |
| Spelling | -0.129 (0.014) | **< 0.001** |
| Writing | -0.105 (0.017) | **< 0.001** |
| **Grade 5 Fall (T3) Course Grades** |  |  |
| Math | -0.095 (0.022) | **< 0.001** |
| Reading | -0.132 (0.018) | **< 0.001** |
| Spelling | -0.138 (0.016) | **< 0.001** |
| Writing | -0.105 (0.019) | **< 0.001** |
| **Grade 4 mean (T1 and T2) daily MVPA and Grade 4 academic achievement, unadjusted results^a^** | | |
| **Academic outcome** | **Coefficient (SE)** | **p-value** |
| **Grade 4 Year (Mean T1 and T2) Course Grades** |  |  |
| Math | -0.184 (0.022) | **< 0.001** |
| Reading | -0.195 (0.018) | **< 0.001** |
| Spelling | -0.184 (0.016) | **< 0.001** |
| Writing | -0.160 (0.020) | **< 0.001** |
| **Grade 4 (T2) Standardized Test Scores** |  |  |
| Math | -0.629 (0.107) | **< 0.001** |
| English Language Arts | -1.017 (0.110) | **< 0.001** |
| Lexile | -3.382 (0.447) | **< 0.001** |
| **Residualized change in course grades from Grade 4 to Grade 5 fall (T3) predicted by Grade 4 mean (T1 and T2) daily MVPA, unadjusted results^a^** | | |
| **Academic outcome** | **Coefficient (SE)** | **p-value** |
| **Grade 5 Fall (T3) Course Grades** |  |  |
| Math | -0.147 (0.023) | **< 0.001** |
| Reading | -0.175 (0.018) | **< 0.001** |
| Spelling | -0.172 (0.016) | **< 0.001** |
| Writing | -0.114 (0.019) | **< 0.001** |

^a^In bold are the p-values that were statistically significant after comparing to a Bonferroni adjusted p-critical of 0.00271.
